# Photoendosymbiosis of the Blue Subtropical *Montipora* Corals of Norfolk Island, South Pacific

**DOI:** 10.3390/microorganisms13092155

**Published:** 2025-09-16

**Authors:** Sophie Vuleta, William P. Leggat, Tracy D. Ainsworth

**Affiliations:** 1 Centre for Marine Science and Innovation, School of Biological, Earth and Environmental Sciences (BEES), The University of New South Wales, Sydney, NSW 2033, Australia; s.vuleta@unsw.edu.au; 2 School of Environmental and Life Sciences, University of Newcastle, Chittaway Road, Newcastle, NSW 2308, Australia; bill.leggat@newcastle.edu.au

**Keywords:** coral, coral reefs, symbiosis, photoendosymbiosis, facultative symbiosis, subtropical, high latitude, environmental factors

## Abstract

Corals exhibit complex and diverse relationships with dinoflagellates of the family Symbiodiniaceae. Montiporid corals within Norfolk Island’s shallow water lagoonal reef systems have been observed to turn a deep fluorescent blue during winter, suggesting potential environmentally driven changes to their photoendosymbiosis. Here, we investigate the photoendosymbiosis of blue *Montipora* sp. corals over a year-long study, demonstrating that photosynthetic yield and Symbiodiniaceae densities vary seasonally, with the lowest photosynthetic yield occurring within winter periods. We also provide the first characterisation of Symbiodiniaceae species associated with corals from Norfolk Island, identifying blue *Montipora* sp. as predominantly associating with *Cladocopium* (formerly Clade C) genotypes (*C3aap*, *C3ig*, and *C3aao*). Finally, we also report on the impact of recent bleaching conditions (March 2024) on blue *Montipora* sp. photoendosymbiosis and find the genera is susceptible to increasing sea surface temperatures. Our findings provide insight into the unique biology of subtropical corals within this remote reef and the susceptibility of corals in the region to increasing sea surface temperatures.

## 1. Introduction

Subtropical reef systems are important ecological zones that encompass unique diversity [1], provide essential ecosystem services [2], and act as critical transition zones between tropical and temperate marine environments, but are understudied [3]. Subtropical coral assemblages remain largely unexplored despite an abundance of undescribed and endemic species [4,5], leaving significant gaps in our understanding of the biodiversity and function of these ecosystems. Predictions of subtropical reef structure and resilience in the face of climate change are therefore uncertain, with limited evidence to support hypotheses regarding the future of these reefs under climate change. In this study, we focus on the major habitat-forming genera *Montipora* within the Norfolk Marine Park and specifically investigate the blue morphotype which exhibits an apparent seasonal flexibility in photoendosymbiosis.

Approximately half of scleractinian coral species maintain an obligate symbiotic relationship with photosynthetic dinoflagellates of the family Symbiodiniaceae, which reside within the coral’s gastrodermal tissue layer (ref. *photoendosymbiosis*) [6,7]. Through photosynthesis, Symbiodiniaceae supply the coral host with the majority of its metabolic energy, enabling the maintenance of homeostasis [8]. Anthropogenic climate change has led to increasingly frequent and severe marine heatwaves, which disrupt photoendosymbiosis, resulting in the phenomena known as coral bleaching. As mass bleaching events (those occurring globally) intensify, there is a growing urgency to improve our understanding of coral–symbiont dynamics, including the diversity, specificity, and flexibility of the symbioses across environmental gradients [9,10]. While tropical reefs have been the primary focus of such research, less is known about symbiotic associations in many subtropical coral reef systems [10], despite their risk of significant population shifts and ecological change [11,12]. For instance, studies from the subtropical Lord Howe Island (the southernmost coral reef in the Pacific) have revealed high levels of symbiont diversity and regional endemism [13], underscoring the ecological uniqueness and research value of subtropical reefs. Investigating symbiotic variability in these marginal environments can offer valuable insights as to how these species may respond to climate change [14]. Given the diverse and complex nature of the coral-Symbiodiniaceae relationship, both between and within coral species [9,15], it is crucial to characterise species-specific symbioses to better understand how coral species, especially key habitat formers, will respond to changing environmental conditions [9,14,16].

Corals exhibiting facultative photoendosymbiosis are valuable models for studying symbiosis as their dependence on Symbiodiniaceae can vary, with the coral host occurring both with and without Symbiodiniaceae. The facultative relationship can fluctuate over time and in response to environmental conditions, such as seasonal change. Notably, facultative corals are predominantly reported in sub-tropical or temperate regions, which remain understudied [15,17,18]. Further, their flexible relationship with Symbiodiniaceae has resulted in facultative species becoming ideal organisms for experimental studies on coral bleaching, adaptation, and resilience, whilst providing deeper insights into the dynamics of the coral-Symbiodiniaceae endosymbiosis in response to environmental influences [19,20]. For example, the well-studied facultative species *Astrangia poculata* have pronounced seasonal fluctuations in photoendosymbiont density and photophysiology, driven by temperature variations characteristic of the subtropical and temperate environments they inhabit [15,17]. Species with facultative photoendosymbiosis are rarely reported, with only 12 coral species identified to date as facultative [21,22], however species-specific photoendosymbiosis in subtropical corals remains poorly characterised. The underexplored coral diversity of subtropical systems therefore provides a valuable opportunity to advance our understanding of species-specific coral–Symbiodiniaceae symbioses, including assessments of temporal and environmental fluctuations in photophysiology and photoendosymbiont densities. Investigating the temporal fluctuations of Symbiodiniaceae densities and photophysiology also provides a better understanding of differing coral biology.

Norfolk Island is a subtropical coral reef system characterised by unique species diversity and estimated high levels of species endemism, yet it has received little scientific attention to date [23,24,25]. Located approximately 1400 km east of the Australian mainland and about 1600 km southeast of the southern edge of the Great Barrier Reef, Norfolk Island is one of Australia’s most remote coral reef ecosystems [24,25]. The Norfolk Marine Park, encompassing 188,000 square km, includes the Norfolk volcanic rise and extends to deep-water habitats reaching depths of up to 5000 m [25]. It is bordered to the north by Australia’s Coral Sea Marine Park, to the west by Australia’s Temperate East Marine Park, and is adjacent to marine parks managed by New Caledonia and New Zealand to the west and south, respectively. Additionally, Norfolk Island lies roughly 800 km northeast of Lord Howe Island, which hosts the world’s southern-most coral reef ecosystem. Norfolk Island is the summit of the Norfolk Rise, with the Norfolk Marine Park encompassing both deep-water habitats and the inshore lagoonal reefs of Emily Bay, Slaughter Bay, and Cemetery Bay [25]. These reef systems host diverse and unique coral assemblages and are hypothesised to exhibit high levels of species endemism [25,26].

In recent years, studies have begun to investigate the ecology and biology of corals within the Norfolk Island reef ecosystem [23,26,27,28]. The genera *Acropora* and *Montipora* are the major reef-building genera within the shallow water lagoonal reef systems [25], however a large percentage of the coral species remain undescribed, as does their photoendosymbioses. One species within the genus *Montipora* has been locally reported to fluoresce blue annually, transitioning from a deep brown colouration in summer and autumn months to bright blue-purple in winter to spring. Coral colony fluorescence has previously been associated with upregulation in photoprotective pigments in the coral host and shifts in photoendosymbiotic densities in response to anomalous environmental conditions [29]. As such, these colour changes potentially indicate seasonal variability in photoendosymbiosis for the host *Montipora* species, making it a unique case study for potential facultative photoendosymbiosis within this subtropical coral reef ecosystem.

Here we aim to describe the photoendosymbiosis of a key habitat-forming coral species within the genus *Montipora*, in Norfolk Island’s subtropical lagoonal coral reef systems, by investigating changes in photoendosymbiosis associated with seasonal variation in colony colouration and fluorescence. We aim to do this by (1) monitoring the colouration of GPS-marked coral colonies to assess temporal changes, (2) investigating the photosynthetic efficiency and photoendosymbiont densities of GPS-located *Montipora* colonies at four timepoints corresponding to lagoonal temperature variations (September 2022, December 2022, April 2023, and August 2023), (3) characterising the photoendosymbiont community by sequencing the nrDNA ITS2 region of tagged colonies across the study period, and (4) investigating the effects of a recent heat stress event on the photoendosymbiotic function and symbiont densities of colonies, relative to other sampling timepoints. To our knowledge, this study represents the first characterisation of the coral—Symbiodiniaceae endosymbiosis on Norfolk Island.

## 2. Materials and Methods

### 2.1. Study Location

**Figure 1:**
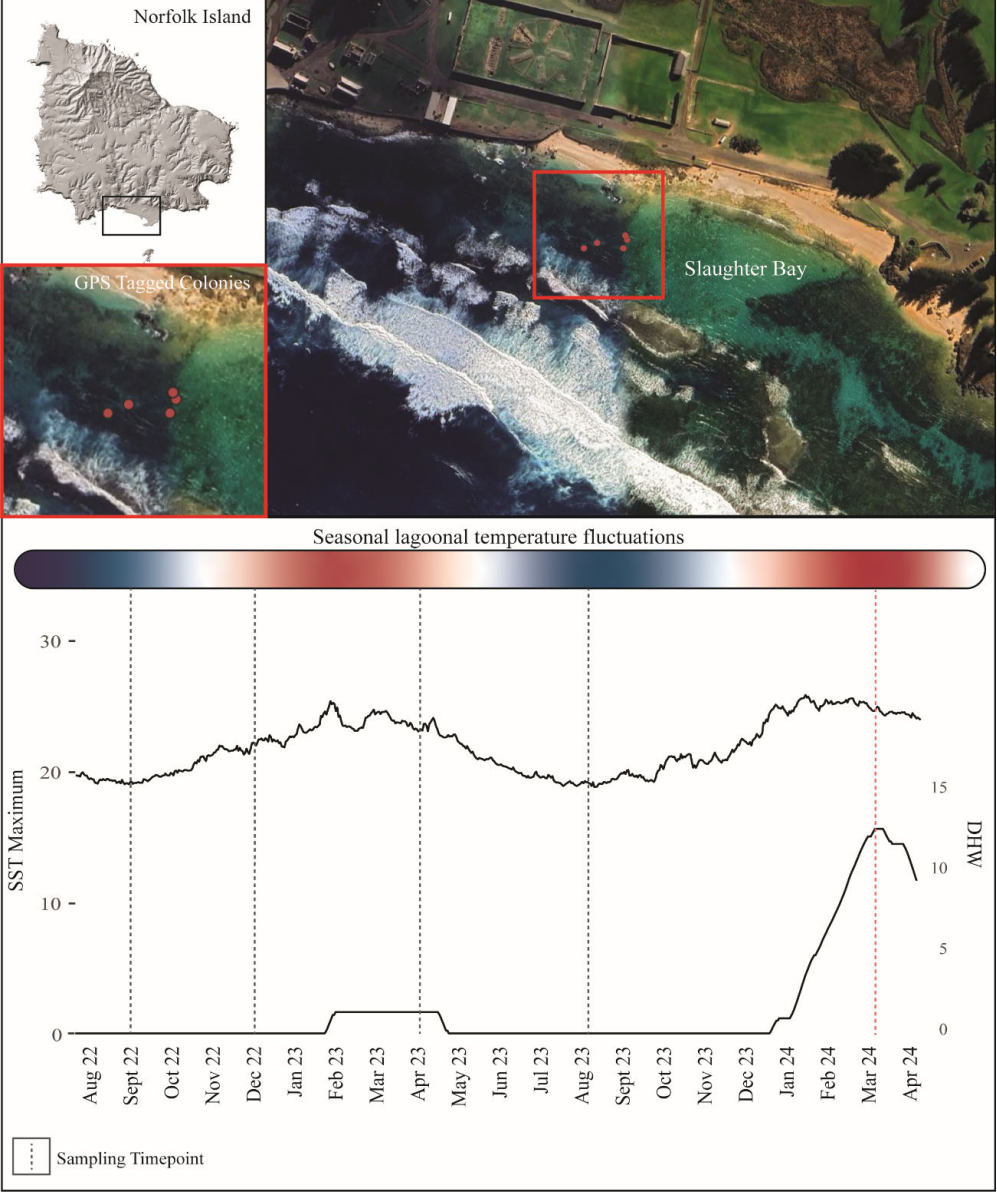
A map of Norfolk Island and an aerial image of the shallow-water lagoonal reef systems. The study site, Slaughter Bay, is indicated on the aerial image and the survey area is outlined in red. The GPS tagged *Montipora* sp. colonies targeted in this study are indicated by red dots. Environmental data, including sea surface temperatures and degree heating weeks, are shown in line graphs. Sampling timepoints are indicated by dashed lines, with the March 2024 bleaching event highlighted in red. Seasonal lagoonal temperature fluctuations are visualised within the heat bar above, with winter and transitional months indicated in blue—white and summer months indicated in red.

This study was based within the shallow-water (<5 m) lagoonal reefs of the Norfolk Island Marine Park (Slaughter Bay, 29°3.5454′ S, 167°57.4434′ E) (Figure 1). Environmental data, including sea surface temperatures, were obtained from the National Oceanic and Atmospheric Administration (NOAA, 2024 [30]) satellite data (Table S1).

### 2.2. Ecological Surveys and Sampling

Five blue *Montipora* sp. (species undescribed (personal communications; Baird, 2024 [31])) colonies were GPS located (Table S2) within Slaughter Bay, September 2022 (<5 m depth). The same colonies were sampled at each timepoint over a 12-month period (September 2022, December 2022, April 2023, and August 2023) and imaged against a CoralWatch coral colour health chart [32]. One sample was collected from the plate edge of each coral colony at each timepoint. Samples were taken from the edge of the colony and were <7 cm in size, not including growth margins which were removed (as indicated by paling, approximately 2 cm). Samples were preserved in 4% paraformaldehyde in sterile phosphate-buffered saline (3XPBS) (PBS; P4417, Sigma-Aldrich, St. Louis, MO, USA) (PFA; C004, Proscitech, Kirwan, Queensland, Australia) solution for 24 h at 4 °C, then transferred to 3XPBS solution and held at 4 °C until analyses.

### 2.3. PAM Fluorometry

Coral samples (n = 5 per timepoint) were dark adapted for 30 min and maximum dark-adapted quantum yield (Fv/Fm)) was analysed using an imaging PAM fluorometer with a MAXI head (Waltz, Effeltrich, Germany). The Minimum fluorescence (Fo) was measured first via exposure to initial weak light pulses, followed by saturating pulses (μmol m^−2^ s^−1^ of PAR for 0.8 s) to measure the maximal fluorescence (Fm). The variable fluorescence yield (Fm-Fo) was then used to calculate the dark-adapted maximum quantum yield (Fv/Fm) reported here. Data was collated in Excel and analysed in R Studio (version 4.2.1) (Tables S1–S3).

### 2.4. Symbiodiniaceae Counts for Density

Tissue was aspirated from coral colony plate samples (n = 5 per timepoint) by water piking (Waterpik Nano, Fort Collins, CO, USA) off the coral skeleton from the top surface of the coral plate. The tissue samples were then washed in water and centrifuged, with excess water discarded and the final tissue pellet re-suspended in 2 mL of milli-Q water. Symbiodiniaceae cells were manually counted for density by placing 20 µL of tissue slurry within a hemocytometer from 3 samples per timepoint (Tables S1–S3). Following replicate counts, total Symbiodiniaceae counts were calculated, taking into account the 2 mL resuspension of cells (Average cells counted × 10,000/1 × 2). The surface area of each sample was then determined following methods outlined by Stimson and Kinzie (1991) and Stimson (1997) using paraffin wax [33,34]. Paraffin wax was heated to 59 °C [34] and coral samples were weighed prior to wax dipping. The top surface of *Montipora* sp. skeletons was then dipped in the paraffin wax and held at an angle after submersion for excess wax to run off, and any drips were discarded. Samples were given 1 min to dry before weighing. The process was then repeated for the second wax dip with results entered in Excel. Eight cylinders of known surface area were used as standards and wax dipped following the same methods. These standards were then used to obtain the equation (y = 0.0303x − 0.127) needed to calculate sample surface area. Surface area was calculated (Sample Surface Area = ((Weight 2 − Weight 1) + 0.127)/0.0303). Symbiodiniaceae cells per cm^2^ were then able to be calculated (Algal Abundance/cm^2^ = Total Symbiodiniaceae Counts/Surface Area). Data were collected in Excel and analysed in R Studio (Tables S1–S3).

### 2.5. DNA Extraction, PCR and Sequencing of the ITS2 Region

DNA was extracted from tissue samples (n = 5 per timepoint) following manufacturer’s instructions of the Invitrogen Recoverall Total Nucleic Acid Isolation Kit for FFPE (Thermofisher Scientific; AM1975, Waltham, MA, USA). Approximately 500 µL aliquots of tissue slurry were placed in 2 mL tubes for DNA extraction. Samples were then centrifuged, with excess water removed prior to digestion. 200 µL of digestion buffer and 4 µL of protease K were then added to the sample. Using 2 matrix M beads, the samples were bead beat for 6 m/s for 30 s and a total of 3 cycles. Following this, samples were left to digest at 56 degrees and 350 rpm rotation for 3 h (digital shaking drybath, Thermo Scientific). The following extraction protocol was in line with the manufacturer’s instructions (see Supplementary Material S1 and S2), until elution. 60 µL of elution solution was added to the filter membrane and left to sit at room temperature for 20–30 min. Samples were centrifuged for 3 min at 14,000 rcf to obtain the final DNA sample. DNA extract was stored at −20 °C until PCR. PCR was conducted in 50 µL reactions using 48 µL master mix and 2 µL of DNA extract, with reagents and volumes outlined in Table 1. Primers used to amplify the internal transcribed spacer 2 (ITS2) nrDNA region were specific to Symbiodiniaceae (SYM_VAR_5.8SII/SYM_VAR_REV [35]). PCR was run in the Eppendorf Mastercycler X50i for 35 cycles with settings outlined in Table 2. A 2% agarose gel (50 mL 1×TAE buffer + 1 g agarose powder + 5 µL GelRed Nucleic acid stain) (30 min at 130 v) was used to check the amplification of the ITS2 region of symbionts against a ThermoScientific 1 kb Plus Generuler. The PCR product was submitted for sequencing at the Ramaciotti Centre for Genomics, within the University of New South Wales, where samples were run on a DNA gel for initial quality control checks, and subsequently cleaned using AMPure Magnetic beads (Beckman Coulter, Brea, CA, USA). Indexing PCR was conducted using Nextera compatible unique dual indexes from Integrated DNA Technologies. The PCR product was then run on a DNA gel for quality control and normalised using the SequalPrep^TM^ Normalisation Plate Kit, 96-well (Thermofisher Scientific, Waltham, MA, USA), with samples pooled together at equal volume and cleaned with AMPure magnetic beads. The final clean pool was quantified using qubit and tapestation before sequencing on a Miseq v2 Nano 2 × 250 bp sequencing run. The ITS2 (Symbiodiniaceae) .fastq files generated from Ramaciotti were then submitted to Symportal to be run remotely through their analytical framework, whereby recurring ITS2 sequences were identified and used to define the ITS2 type profile. Postmed sequencing relative abundance files were transferred to Excel and analysed in R Studio (version 4.2.1) (Supplementary Material S3).

### 2.6. Statistical Analyses

PAM data, Symbiodiniaceae density data and ITS2 data were analysed in R Studio (version 4.2.1) using ANOVA analyses and Tukey post hoc testing where applicable. All code and statistics are available via OSF (Supplementary Material S4).

## 3. Results

### 3.1. Ecological Observations

**Figure 2:**
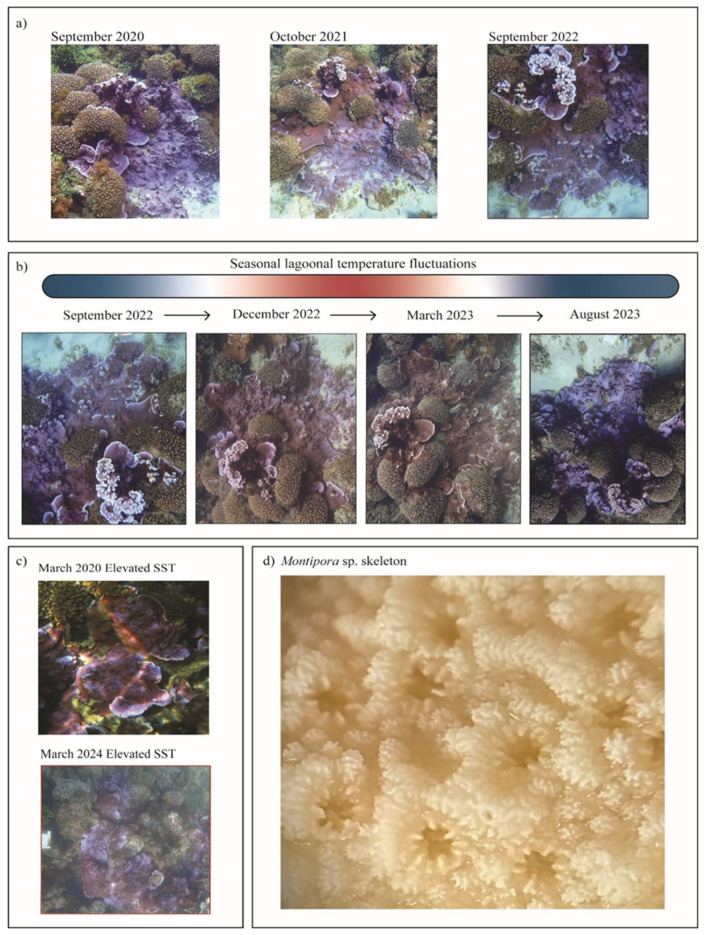
(**a**) Historic images of blue *Montipora* sp. from September 2020, October 2021, and September 2022 (images by Susan Prior, with permission). (**b**) *Montipora* sp. imaged over 4 timepoints within this study demonstrating colour variation with seasonality, with temperature fluctuations visualised in the heat bar above. (**c**) Images of *Montipora* sp. taken within the March 2024 bleaching event in Norfolk Island’s lagoonal reef system, demonstrating patchy fluorescence. A similar response is observed in photos obtained from March 2020 under elevated SST (images by Susan Prior, with permission). (**d**) A skeletal image of blue *Monitpora* sp.

All 5 coral colonies of *Montipora* sp. (Figure 2) assessed in September 2022 and August 2023 were found to demonstrate blue, fluorescent colouration across the whole colony (Figure 2). All 5 colonies were observed to darken to a brown colouration across the whole colony from December 2022 to April 2023 (Figure 2).

### 3.2. PAM Fluorometry and Symbiodiniaceae Counts for Density

**Figure 3:**
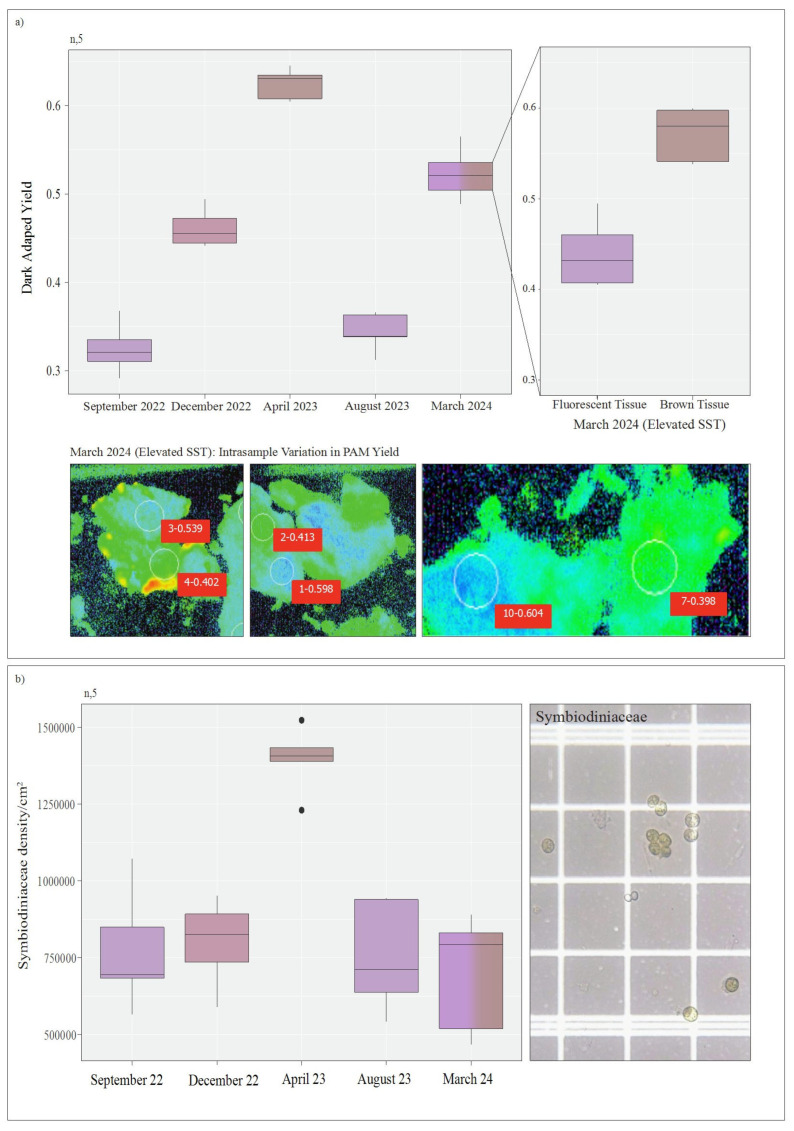
(**a**) Dark adapted pulse amplitude modulation fluorometry of *Montipora* sp. within Norfolk Island’s lagoonal reef across 5 timepoints, including a bleaching event in March 2024 indicated in red. *Montipora* sp. demonstrated significant variation in photosynthetic yield over time, with yield decreasing in the winter or spring transitional months and increasing into late summer. *Montipora* sp. had a patchy bleaching response in March 2024 (i.e., patches of fluorescent tissue and brown tissue) which resulted in significant variation in photosynthetic yield across the colony. (**b**) Symbiodiniaceae densities were observed to significantly increase in April 2023. The March 2024 bleaching event demonstrated comparatively lower Symbiodiniaceae densities when compared to previous similar timepoints (April 2024).

Dark adapted yield was found to be significantly different across all timepoints (df = 3, F value = 50.48, *p* = 2.22 × 10^−8^ except September 2022 and August 2023 (*p* = 0.999) (Figure 3), which both represented periods of decreased temperature within the lagoonal system (Figure 1). September 2022 samples averaged a dark-adapted yield of 0.322 (±0.027). December samples averaged a dark-adapted yield of 0.508 (±0.087) representing a 36.6% increase in yield from September. In April, sampled colonies averaged 0.634 (±0.020) dark adapted yield, illustrating a 19.8% increase from December, and August samples averaged 0.318 (±0.025) dark adapted yield representing a 49.9% decrease in dark-adapted yield from April. During March 2024 (elevated SST conditions) samples averaged a dark-adapted yield of 0.523 (±0.013) using the whole sample (encompassing both fluorescent and brown patches of tissue). However, samples demonstrated further decreased dark-adapted yield within patches of high fluorescence (averaging 0.0440 (±0.013) in patches of fluorescence and 0.571 (±0.017) on browner tissue, exhibiting an average yield variance of 23% across the sample).

Symbiodiniaceae densities within April 2023, representing the period of highest lagoonal sea surface temperatures within the study (Figure 2), were found to be significantly different (df = 3, F value = 19, *p* = 1.59 × 10^−5^) from all other timepoints, reporting the highest symbiont densities (averaging 1,396,720 per cm^2^) (Figure 3). No significant differences were found between the remaining timepoints. A 42.8% increase in photoendosymbiont density was further reported between samples taken in December 2022 and April 2023, followed by a 46% reduction in symbiont density for samples obtained in August 2023.

### 3.3. Characterisation of the Symbiodiniaceae Community

**Figure 4:**
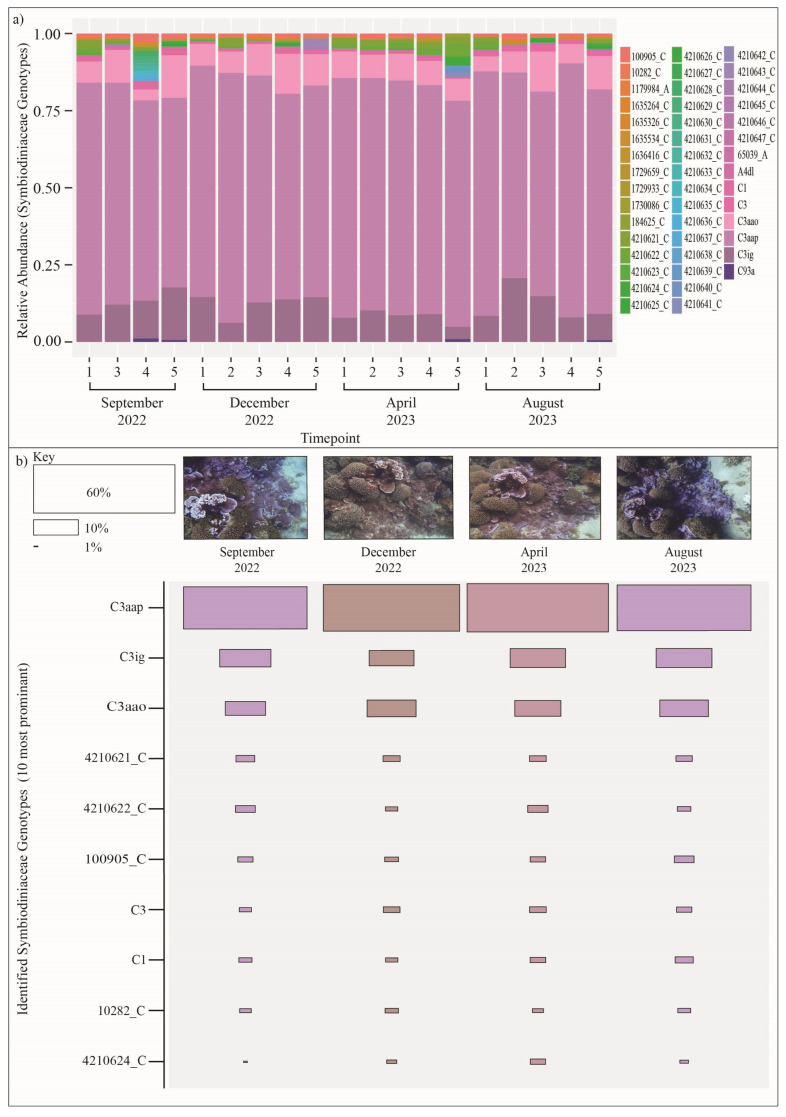
(**a**) Symbiodiniaceae genotypes identified for *Montipora* sp. within Norfolk Island’s lagoonal reefs by targeting internal transcribed spacer 2 (ITS2) nuclear ribosomal DNA region using Symbiodiniaceae specific primers across 4 timepoints. *Montipora* sp. associate with *Cladocopium* genotypes. *C3aap* was the predominant genotype identified followed by *C3ig* and *C3aao*. A large array of less prominent genotypes are also shown in association. (**b**) Colonies are grouped by timepoint to demonstrate minimal variability in the associated genotypes over time.

PCR amplification and sequencing of the ITS2 region of nuclear ribosomal DNA from Symbiodiniaceae isolated from *Montipora* sp. (n,5) across 4 timepoints, found *Montipora* sp. to associate with the Symbiodiniaceae genera *Cladocopium* (formerly referred to as Clade C) (Figure 4). *Cladocopium* genotype *C3aap* was identified as the predominant photoendosymbiont across all colonies at all timepoints, representing approximately 73% of the Symbiodiniaceae population on average (Figure 4). Genotypes reported as *C3ig* and *C3aao* represented approximately 11% and 9% of the photoendosymbiont populations, respectively, with high levels of additional *Cladocopium* diversity also reported in less abundances (≤1%) (Figure 4; Supplemental Material S3). No significant difference (df = 3, F value = 1.455, *p* = 0.226) in the abundance of the predominant photoendosymbiont type was found between sampling timepoints.

## 4. Discussion

This study investigated species-specific photoendosymbiosis in blue morphotypes of *Montipora* sp. corals within Norfolk Island’s shallow water subtropical lagoonal reef system, revealing temporal changes in photoendosymbiosis and highlighting the variability of scleractinian photoendosymbiosis in marginal reef habitats. *Montipora* sp. colonies were observed to change colour seasonally, turning fluorescent blue during the cooler winter and spring months (August and September timepoints) and reverting to a brown pigmentation as lagoonal temperatures increased (December and April timepoints). To determine whether these colour changes reflected shifts in photoendosymbiosis, we assessed Symbiodiniaceae densities and photosynthetic efficiency, both of which varied across timepoints alongside temperature fluctuations. We also characterised the Symbiodiniaceae communities associated with blue *Montipora* sp., finding stable associations with *Cladocopium* genotypes (*C3aap*, *C3ig*, and *C3aao*). This represents the first characterisation of Symbiodiniaceae associated with corals from Norfolk Island’s lagoonal reef system. In addition, we also investigated the effects of a recent bleaching event (occurring from February to March 2024) on photoendosymbiotic function and symbiont density, finding these corals to be susceptible to elevated sea surface temperatures.

Subtropical reef systems experience greater environmental variability than tropical ecosystems, with fluctuations in temperature and irradiance occurring seasonally, which have been shown to promote changes in coral photoendosymbiosis that are not well understood [15,17,36]. An upregulation of fluorescent proteins, evident by bright coloration in corals, usually during summer months, is a strong visual indicator of changes in the coral photoendosymbiosis and has commonly been found to co-occur with the loss of Symbiodiniaceae during coral bleaching [37,38,39,40,41]. However, here the annual occurrence of *Montipora* sp. blue fluorescence within winter periods (reported by personal communication with local reef users), and their return to browner pigmentation as temperatures rise, suggested seasonal changes in photoendosymbiosis, rather than an acute stress response causing a loss of Symbiodiniaceae, which is known to significantly impact colony health, function, and reproduction long-term [42].

*Montipora* sp. were found to exhibit variability in photoendosymbiotic function and symbiont density seasonally within the subtropical lagoonal reef system of Norfolk Island, illustrating the interplay of coral photoendosymbiosis and environmental variability. *Montipora* sp. photosynthetic yield and Symbiodiniaceae densities were found to be significantly lower within the cooler winter or spring transitional months comparative to late summer, demonstrating contrasting photoendosymbiotic patterns to most tropical coral species, which commonly increase Symbiodiniaceae densities into winter [43]. Dimond & Carrington (2007) discuss these inverse trends, which were also identified for the subtropical and temperate facultative species *A. poculata*, as a reflection of corals living at their lower thermal limits (subtropical and temperate regions) [17]. Further, temperate facultative coral species have been shown to reduce Symbiodiniaceae densities into winter, when the photoendosymbiosis offers less benefits to the coral host under conditions promoting reduced photosynthetic productivity [15], resulting in the colony’s increased reliance on heterotrophy. Additionally, under significant temperature declines some scleractinian coral species have also been shown to exhibit winter dormancy, demonstrating reductions in metabolism, growth [44], and tissue thickness [17], with reduced Symbiodiniaceae densities hypothesised to conserve host resources [15]. Together, photophysiology and Symbiodiniaceae density measurements suggest that *Montipora* sp. photoendosymbiosis varies in response to seasonal temperature fluctuations on Norfolk Island, although it remains unclear whether colonies increase their reliance on heterotrophically derived nutrition or enter periods of relative dormancy under reduced temperatures. *Montipora* sp. further present an interesting case study for the investigation of photoendosymbiotic variability encompassed by scleractinian corals throughout different marine habitats.

The breadth of the coral photoendosymbiotic spectrum is not well defined or understood. Whilst species are typically defined as either photoendosymbiotic, facultatively photoendosymbiotic, or aphotoendosymbiotic [7,45], when considered as rigid groupings, these terms fail to capture the extent of variability encompassed within the coral—Symbiodiniaceae relationship. Facultative species, though rare [22], are often used to explore the variability of coral photoendosymbiosis due to their ability to live both with and without Symbiodiniaceae [15,17,22,36]. Research conducted on these species further highlights the influence of environmental factors on coral photoendosymbiosis, including changes in photoendosymbiotic function or density [15,17]. However, some scleractinian coral species, especially those residing within more dynamic environments, such as the *Montipora* sp. discussed here, exhibit more flexible or variable relationships with Symbiodiniaceae, yet they do not fully meet the criteria for being classified as facultative. This highlights a significant research gap in our understanding of the diversity of coral photoendosymbiosis, which is largely shaped by species specificity and biogeography [9]. This further emphasises the importance of characterising species-specific photoendosymbiosis across diverse marine habitats, particularly in subtropical systems, as species may not adhere to the prevailing assumptions of coral photoendosymbiosis which are commonly developed within tropical regions. This presents interesting, fundamental biological differences in photoendosymbiosis across species and habitats, which, when properly investigated, will enhance our understanding of its evolution and adaptation, while also providing insight into broader ecosystem dynamics and their response and management under future environmental change.

*Montipora* sp. were found to be associated with the Symbiodiniaceae genera *Cladocopium* (formerly referred to as Clade C), representing the first characterisation of Symbiodiniaceae within Norfolk Island’s subtropical reef system. The characterisation of the coral—Symbiodiniaceae symbiosis enables insight to into the partnership’s response under changing environmental conditions [46]. Like their coral hosts, Symbiodiniaceae demonstrate significant phylogenetic diversity, and with that, diverse responses to changing temperature regimes [47]. Often, Symbiodiniaceae characterisations within remote subtropical regions are absent from the literature with studies instead focusing on broader ecosystem ecology [48], presenting research gaps in our understanding of the coral—Symbiodiniaceae symbiosis in reef corals residing at their range extents, and difficulty assessing their likely stress response under periods of elevated sea surface temperatures. Here we identified *Montipora* sp. to associate with *Cladocopium*, representing one of the most diverse and wide-spread Symbiodiniaceae genera [49]. In their systematic revision of Symbiodiniaceae, LaJeunesse et al. (2018) [49] report that *Cladocopium* species frequently exhibit host specialisation, while also demonstrating adaptability to a wide range of environmental conditions, such as temperature and irradiance [49], making it common among corals that have been characterised within dynamic subtropical reef systems [10,50,51]. This includes corals within subtropical reefs of the South Pacific specifically, such as Lorde Howe Island, which demonstrated associations with a diverse array of novel *Cladocopium* genotypes [52]. Similarly, *Montipora* sp. here show uncommon predominant *Cladocopium* genotypes (*C3aap*, *C3ig* and *C3aao*) and a high diversity of background *Cladocopium* genotypes.

No significant shifts in the dominant Symbiodiniaceae genotypes for *Montipora* sp. were observed in association with fluctuations in photosynthetic yield or Symbiodiniaceae densities over the 4 timepoints analysed within this study, suggesting a host-specific response to seasonality. Some studies report compositional change of the Symbiodiniaceae population in bleaching and recovery [53,54], with more resilient background types often predominating under or following unfavourable environmental conditions (i.e., elevated SST). However, here we observe, despite an apparent increase in photoendosymbiont densities and photosynthetic yield into summer periods, relative stability of the identified Symbiodiniaceae community composition for *Montipora* sp. colonies. Similarly, the temporal stability of Symbiodiniaceae communities was observed in *Pocillopora damicornis* colonies within the subtropical reefs of Lord Howe Island [13]. Here however, as the photosynthetic efficiency and photoendosymbiont densities of *Cladocopium* spp. significantly varied with seasonality, it is likely *Montipora* sp. exhibit a host-specific response, or regulation, of Symbiodiniaceae with seasonal variations in temperature, as opposed to a Symbiodiniaceae driven response.

*Montipora* sp. were found to exhibit patchy fluorescence, reduced Symbiodiniaceae densities, and reduced photophysiology in response to a recent bleaching event recorded on Norfolk Island (March 2024), highlighting them as vulnerable to the effects of climate change despite their apparent photoendosymbiotic flexibility. Species exhibiting photoendosymbiotic variability have been hypothesised to be more resilient due to their biological adaptations that allow them to endure a wider range of environmental conditions [36]. However, by contrast, these species often reside within high latitude regions which are predicted to undergo significant environmental change [3,12]. Here we find that despite apparent temporal photophysiological fluctuations in response to seasonal temperature variability in the subtropical lagoonal reefs of Norfolk Island, *Montipora* sp. demonstrated an acute stress response to prolonged elevated SST, as evident by patchy fluorescence, reduced photosynthetic yield, and photoendosymbiont densities. Interestingly, encrusting species, such as the montiporid corals found on Norfolk Island, have generally been considered more resilient to thermal stress [55], with some *Montipora* species in particular recognised for their thermal tolerance in tropical environments [56]. However, within the March 2024 bleaching event on Norfolk Island, 6.4% and 41.3% of montiporid species were found to be either bleached or pale, respectively, indicating that bleaching susceptibility patterns may vary into the subtropics. Further, species residing within high latitude environments often alter aspects of their physiology to persist under lower thermal conditions [57] meaning periods of elevated SST still pose significant threats to species within these systems. However, it remains unclear whether species exhibiting variable photoendosymbiosis, such as the *Montipora* sp. presented here, can better withstand and recover from periods of reduced photoendosymbiotic function as a result of their temporally variable relationship with Symbiodiniaceae, which warrants further investigation.

## 5. Conclusions

This study identified seasonal variation in photosynthetic efficiency and Symbiodiniaceae densities for blue *Montipora* sp. colonies within Norfolk Island’s subtropical lagoonal reef systems. These corals also exhibited reduced photosynthetic efficiency and symbiont densities in response to a recent bleaching event (March 2024). We further identified that *Montipora* sp. predominantly hosts Symbiodiniaceae of the genus *Cladocopium* (*C3aao*, *C3ig*, and *C3aap*), representing the first characterisation of Symbiodiniaceae within Norfolk Island’s shallow-water lagoonal reef systems. Notably, we found no evidence of seasonal shifts in the dominant symbiont community composition. Unlike their tropical counterparts, *Montipora* sp. in subtropical reefs should be considered susceptible to rising sea surface temperatures, exhibiting reduced photosynthetic performance and Symbiodiniaceae densities during bleaching events. Our study emphasises the importance of understanding species-specific photoendosymbioses in scleractinian corals across diverse marine habitats to better capture the complexity of coral–Symbiodiniaceae relationships and assess the impacts of rising sea surface temperatures on subtropical reef ecosystems. However, it is important to note that given the remote nature of the site and the fact that this study was limited to a one-year timeframe, further research over an extended time-period would be valuable to more precisely attribute changes in photoendosymbiosis to temperature and light fluctuations linked with seasonality. Further, given the unique biology of corals in the Norfolk Island region, evident by the temporal changes in photoendosymbiosis reported here, further studies are also needed to determine the energetic biology of these corals, such as their potential reliance on heterotrophy and/or bacterial symbioses during declines in photoendosymbiosis. Understanding the energetic and nutritional biology of these corals may provide greater insights into the symbiotic variability of these corals and their potential resilience to increases in sea surface temperatures.

## Figures and Tables

**Table 1:** Reagents and volumes used within PCR.

Reagent	µL
10x PCR buffer (QIAGEN, Hilden, Germany)	5
SYM_VAR_5.8S2 (Forward primer) (Ramaciotti, Sydney, Australia)	1
SYM_VAR_REV (Reverse primer) (Ramaciotti)	1
dNTP Mix, PCR Grade (10 mM each) (QIAGEN)	1
HotStarTaq DNA Polymerase (QIAGEN)	0.5
DNase/RNase free water (Invitrogen, Thermofisher Scientific)	39.5
Extracted DNA (>10 ng/µL)	2

**Table 2:** PCR settings at 35 cycles.

Temperature (°C)	Time
95	15 min
98	10 s
56	30 s
72	1 min
75	1 min
10	Holding temperature

## Data Availability

The data presented in this study are openly available in OSF at https://osf.io/scbe4/?view_only=c00d264a1eb64f8fb41800d846c84579 (accessed on 27 February 2025).

## Supplementary Materials

The following supporting information can be downloaded at: https://www.mdpi.com/article/10.3390/microorganisms13092155/s1, Supplementary Material S1: DNA Extraction Protocol Notes; Supplementary Material S2: DNA Extraction Protocol Recoverall; Supplementary Material S3: ITS2 Data; Supplementary Material S4: Statistics and Code; Table S1: Temperature Data; Table S2: Montipora GPS Coordinates; Table S3: PAM Data; Table S4: PAM Data March; Table S5: Symbiont Counts and Waxing.

## Abbreviations

The following abbreviations are used in this manuscript:

SST	Sea Surface Temperature(s)
PAM	Pulse Amplitude Modulation Fluorometry
