# Supplementary material for: Photoendosymbiosis of the Blue Subtropical Montipora Corals of Norfolk Island, South Pacific
Source: Microorganisms. 2025 Sep 16;13(9):2155. doi: 10.3390/microorganisms13092155 (PMC12472439; doi:10.3390/microorganisms13092155)
Supplement: Supplementary file 1 [file microorganisms-13-02155-s001.zip › Supplementary Material S1 DNA_Extraction_Protocol_Notes.pdf]

DNA Extraction Notes

Take the pellets of *Symbiodiniaceae* cells for DNA extraction.

1) Put the sample in tube with lysis beads of choice (2 matrix M beads) (200 uL Digestion Buffer, 4 uL Proteinase – protocol)
2) Put on the bead beater using the settings of 6m/s for 30 seconds with 3x cycles
3) After bead beating centrifuge it for 4 mins at 5000-6000rpm to get the bubbles out.
4) Pipette out all of the sample into new 2ml tubes to then put on the 56 degree heat block. Try to avoid putting too much of the bubbles into the new tube. On the heat block have samples shake at 350rmp.
5) Put it to digest for 3 hours. (leave it on the heat block until the liquid doesn't look hazy/chunky with particles. I recommend anywhere between 2-3.5 hours depending on your sample).

- While this is happening set up new filter tubes with the spare plastic columns that you will need in the wash steps. You setup 3x columns for each sample, so 8 samples then 24 spare columns for the wash cycles.

6) Take it off digestion and centrifuge it quickly to get drops off the lid.
7) Add 240 uL Isolation additive and 550 uL 100% molec grade ethanol to 2mL tube, and mix (pipette up and down/vortex).
8) Pipette all the sample (580ul of at a time) into the filter cartridge tube (it only fits 580ul of liquid at a time). Centrifuge for 1 min at 8,000rpm making sure all the sample has passed through the filter. If it hasn't then centrifuge again at 10,000rpm for 30 sec. repeat this as many times as needed until all the digested sample has been pipetted through the tube.
9) Discard the liquid and put the filter into a new plastic column. Add 700ul of Wash buffer 1 and incubate at room temperature for 60 seconds. Centrifuge for 30 seconds at 10000 rpm.
10) Add 500 ul of wash buffer 2/3 and centrifuge for 30 seconds at 10000 rpm.
11) Repeat wash buffer 2/3.
12) Put filter into new column and dry the membrane by centrifuging 3mins 14,000rpm.
13) Setup new Eppendorf 2ml tubes for the final DNA to stay in
14) Put the dry filter cartridge into the new Eppendorf tube and add 60ul of elution solution to the center of the filter.
15) Incubate it at room temp with the lid on for 15-30 mins before centrifuging for 3 mins 14,000rpm.
