# Supplementary material for: Photoendosymbiosis of the Blue Subtropical Montipora Corals of Norfolk Island, South Pacific
Source: Microorganisms. 2025 Sep 16;13(9):2155. doi: 10.3390/microorganisms13092155 (PMC12472439; doi:10.3390/microorganisms13092155)
Supplement: Supplementary file 1 [file microorganisms-13-02155-s001.zip › Supplementary Material S2 DNA_Extraction_Protocol_Recoverall.pdf]

PROTOCOL

ambion® | RNA
by life technologies™

RecoverAll™ Total Nucleic Acid Isolation Kit

life technologies™

For Research Use Only. Not intended for any animal or human therapeutic or diagnostic use.

Information in this document is subject to change without notice.

APPLIED BIOSYSTEMS DISCLAIMS ALL WARRANTIES WITH RESPECT TO THIS DOCUMENT, EXPRESSED OR IMPLIED, INCLUDING BUT NOT LIMITED TO THOSE OF MERCHANTABILITY OR FITNESS FOR A PARTICULAR PURPOSE. TO THE FULLEST EXTENT ALLOWED BY LAW, IN NO EVENT SHALL APPLIED BIOSYSTEMS BE LIABLE, WHETHER IN CONTRACT, TORT, WARRANTY, OR UNDER ANY STATUTE OR ON ANY OTHER BASIS FOR SPECIAL, INCIDENTAL, INDIRECT, PUNITIVE, MULTIPLE OR CONSEQUENTIAL DAMAGES IN CONNECTION WITH OR ARISING FROM THIS DOCUMENT, INCLUDING BUT NOT LIMITED TO THE USE THEREOF, WHETHER OR NOT FORESEEABLE AND WHETHER OR NOT APPLIED BIOSYSTEMS IS ADVISED OF THE POSSIBILITY OF SUCH DAMAGES.

LIMITED USE LABEL LICENSE: RESEARCH USE ONLY
The purchase of this product conveys to the purchaser the limited, non-transferable right to use the purchased amount of the product only to perform internal research for the sole benefit of the purchaser. No right to resell this product or any of its components is conveyed expressly, by implication, or by estoppel. This product is for internal research purposes only and is not for use in commercial applications of any kind, including, without limitation, quality control and commercial services such as reporting the results of purchaser's activities for a fee or other form of consideration. For information on obtaining additional rights, please contact **outlicensing@lifetech.com** or Out Licensing, Life Technologies, 5791 Van Allen Way, Carlsbad, California 92008.

TRADEMARKS

The trademarks mentioned herein are the property of Life Technologies Corporation or their respective owners.

TaqMan is a registered trademark of Roche Molecular Systems.

© 2011 Life Technologies Corporation. All rights reserved.

Part Number 1975M Rev. C
02/2011

RecoverAll™ Total Nucleic Acid Isolation Kit

(Part Number AM1975)

Protocol

I. Introduction 1

A. Product Description
B. Background
C. Procedure Overview
D. Materials Provided with the Kit and Storage Conditions
E. Materials Not Provided with the Kit
F. Related Products

II. RecoverAll Total Nucleic Acid Isolation Protocol 7

A. Before You Begin
B. Deparaffinization
C. Protease Digestion
D. Nucleic Acid Isolation
E. Nuclease Digestion and Final Nucleic Acid Purification

III. Assessing Nucleic Acid Yield and Integrity 15

A. Factors Affecting Nucleic Acid Recovery and Integrity
B. Assessing RNA Yield and Integrity
C. DNA Yield and Quality

IV. Troubleshooting 20

A. Nucleic Acid Appears Fragmented
B. Yield of Nucleic Acid Is Low
C. Nucleic Acid Inhibits Enzymatic Reactions; Contaminants in Nucleic Acid

V. Appendix 22

A. References
B. Quality Control
C. Safety Information

VI. Documentation and Support 25

A. Obtaining SDSs
B. Obtaining support

I. Introduction

IMPORTANT

Before using this product, read and understand the "Safety Information" in the appendix in this document.

A. Product Description

The RecoverAll™ Total Nucleic Acid Isolation Kit is designed to extract total nucleic acids (RNA, miRNA, and DNA) from formaldehyde- or paraformaldehyde-fixed, paraffin-embedded (FFPE) tissues. Up to four 20 μm sections, or up to 35 mg of unsectioned core samples, can be processed per reaction.

The RecoverAll advantage

The RecoverAll Total Nucleic Acid Isolation procedure requires about 45 minutes of hands-on time and can easily be completed in less than one day when isolating RNA (see Figure 1 on page 3). FFPE samples are deparaffinized using a series of xylene and ethanol washes. Next, they are subjected to a rigorous protease digestion with an incubation time tailored for recovery of either RNA or DNA. Nucleic acids are purified using a rapid glass-fiber filter methodology that includes an on-filter nuclease treatment and are eluted into either water or the low salt buffer provided.

The recovered nucleic acids are suitable for downstream applications such as microarray analysis, quantitative real-time RT-PCR, and mutation screening. However, sample fixation and storage typically cause nucleic acid fragmentation and modification. The RecoverAll procedure does not affect nucleic acid fragmentation, and some chemical modifications may remain after the procedure, therefore some downstream procedures may require modification for best results.

B. Background

Is RNA recovered from paraffin-embedded samples suitable for analysis?

The ability to isolate nucleic acid that is suitable for molecular analysis from archived tissue samples provides a powerful tool in retrospective studies of diseased tissue at both the genomic and gene expression level. Standard preservation techniques for storage of biological tissue samples use formaldehyde or, less frequently, paraformaldehyde. The high reactivity of these chemicals makes them ideal for maintaining tissue structure and preventing putrefaction, however, tissues preserved in this fashion have historically been thought to be unusable for molecular analysis. Nucleic acid is both trapped and modified by extensive protein-protein and protein-nucleic acid crosslinks. RNA (and DNA) in histological samples typically is fragmented and chemically modified to a degree that renders it incompatible with many molecular analysis techniques.

Interestingly, we and others (Masuda 1999) have found that the fixation process itself does not necessarily lead to RNA fragmentation. In fact, fairly large RNA molecules can be extracted from samples that have been stored in buffered formalin (NBF) for up to several months. During standard embedding processes, however, high temperatures required for paraffin infiltration can accelerate chemical reactions which modify RNA and DNA. During storage, these modifications can cause nucleic acid fragmentation, especially of RNA.

Tissue samples that have been fixed and embedded in paraffin using standard protocols, and subsequently stored for only weeks or months, can provide relatively large (although modified) RNA species. Nucleic acid degradation continues during storage in paraffin, so that samples that have been stored over a decade typically yield nucleic acids only in the ~100 nt range. However, even these samples can be analyzed by quantitative real-time RT-PCR if a small (<100 bp) amplicon is chosen (Specht 2001 and Cronin 2004).

The degree of RNA fragmentation that has already occurred in FFPE samples prior to nucleic acid isolation cannot be changed. However, the protease digestion conditions of the RecoverAll Total Nucleic Acid Isolation Kit are designed to release, in a relatively short period of time (30 min), a maximal amount of RNA fragments of all sizes, including miRNA. The recovered RNA can be used in real-time RT-PCR. With allowances for the inevitable 3' end bias, some samples are amenable to array analysis after amplification using, for example, the MessageAmp™ III or MessageAmp™ Premier Kits (P/N AM1793, AM1792, respectively); see page 18 for further discussion.

What about DNA?

DNA tends not to fragment as easily as RNA. However, the nucleo-histone matrix is quite dense and also appears to be much more reactive to formaldehyde. A much longer protease digestion time (overnight instead of 30 min) is required to release substantial amounts of DNA from the tissue extract. After purification on the glass-fiber filter, DNA recovered with the RecoverAll Total Nucleic Acid Isolation Kit can typically be used for PCR and other downstream applications.

C. Procedure Overview

Paraffin-embedded samples are incubated in xylene at elevated temperatures to solubilize and remove paraffin from the tissue, then washed in alcohol solutions to remove the xylene. If recovery of miRNA is desired, we recommend using slices that are ≥10 μm in thickness. The deparaffinized samples are next subjected to a protease step to digest protein covalently bound to RNA, DNA, and other protein. Finally, the nucleic

Figure 1. Overview of the RecoverAll Total Nucleic Acid Isolation Procedure

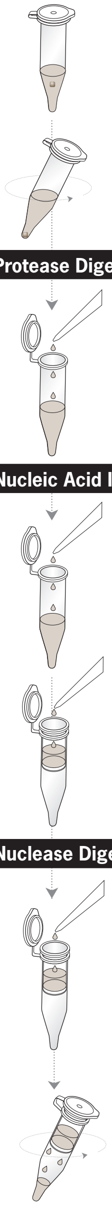

Deparaffinization

20 min

1. Assemble FFPE sections equivalent to ≤ 80 µm or ≤ 35 mg unsectioned core
2. Add 1 mL 100% xylene, mix, and incubate for 3 min at 50°C
3. Centrifuge for 2 min at maximum speed, and discard the xylene
4. Wash the pellet twice with 1 mL 100% ethanol
5. Vacuum or air dry the pellet to remove residual ethanol

Protease Digestion

30 min or 16 hr

1. Add Digestion Buffer and Protease
2. Incubate at 50°C/80°C (RNA) or at 50°C (DNA)

Nucleic Acid Isolation

10 min

1. Prepare Isolation Additive/ethanol mixture
2. Add Isolation Additive/ethanol and mix
3. Pass the mixture through a Filter Cartridge
4. Wash with 700 µL of Wash 1
5. Wash with 500 µL of Wash 2/3, and then centrifuge to remove residual fluid

Nuclease Digestion and Final Purification

45 min

1. Add DNase or RNase mix to each Filter Cartridge and incubate for 30 min
2. Wash with 700 µL of Wash 1
3. Wash twice with 500 µL of Wash 2/3, then centrifuge to remove residual fluid
4. Elute with 60 µL Elution Solution or nuclease-free water at room temp (RNA) or 95°C (DNA)

acid of choice is purified by capture on a glass-fiber filter, washing and elution. High-ethanol washing steps ensure the recovery of smaller RNA fragments (<200 nt), including miRNA.

D. Materials Provided with the Kit and Storage Conditions

This kit contains reagents for 40 isolations of total RNA or DNA from paraffin-embedded tissue.

Amount	Component	Storage
16 mL	Digestion Buffer	room temp
60 mL	Wash 1 Concentrate<br>Add 42 mL 100% ethanol before use	room temp
60 mL	Wash 2/3 Concentrate<br>Add 48 mL 100% ethanol before use	room temp
80	Collection Tubes	room temp
40	Filter Cartridges	room temp
19.2 mL	Isolation Additive	room temp
5 mL	Elution Solution	any temp*
160 µL	Protease	–20°C
240 µL	10X DNase Buffer	–20°C
160 µL	DNase	–20°C
400 µL	RNase A	–20°C

* Store Elution Solution at –20°C, 4°C, or room temp.

Note that the kit is shipped in two parts: one at room temperature and the second at –20°C.

E. Materials Not Provided with the Kit

Reagents and equipment

- 100% xylene, ACS grade or higher quality
- 100% ethanol, ACS grade or higher quality
- Microtome for tissue sectioning
- (Optional) Equipment for cutting, and grinding or crushing, unsectioned core samples [e.g., Harris Micro-Punch™, small mortar and pestle (preferably polished agate), and liquid N_2]
- 1.5 mL microcentrifuge tubes (e.g., P/N AM12400, AM12450; for initial digestion)—these do not have to be nuclease-free
- Adjustable pipettors and RNase-free tips
- Microcentrifuge capable of at least 10,000 x g
- Incubators or heat blocks (deep well preferred) set at 50°C, 80°C, and 95°C

- (Optional) Non-stick tubes (e.g., P/N AM12450), for long-term storage of recovered nucleic acid
- (Optional) Centrifugal vacuum concentrator (e.g., SpeedVac), for drying deparaffinized tissue samples

For RNA or DNA analysis

- Spectrophotometer, e.g., the NanoDrop® 1000 Spectrophotometer
- Agilent® 2100 bioanalyzer, or reagents and apparatus for preparation and electrophoresis of agarose gels
- (Optional) TaqMan® RNase P Detection Reagents Kit (FAM™) (e.g., P/N 4316831) and TaqMan® DNA Template Reagents (e.g., P/N 401970), for quantifying DNA using real-time PCR

F. Related Products

RNase*Zap*® Solution<br>P/N AM9780–AM9784	RNase Decontamination Solution. RNase*Zap* is simply sprayed or poured onto surfaces to instantly inactivate RNases. Rinsing twice with distilled water will eliminate all traces of RNase and RNase*Zap*.
TURBO™ DNase Reagent<br>P/N AM2238, AM2239	TURBO DNase is a hyperactive DNase that was developed using a protein engineering approach to improve wild-type DNase I. These changes markedly increase the affinity of the protein for DNA. The result is a versatile enzyme with a 6-fold lower K_m for DNA, and an ability to maintain at least 50% of peak activity in solutions approaching 200 mM monovalent salt, even when the DNA concentration is in the nanomolar (nM) range.
TURBO DNA-*free*™ Kit<br>P/N AM1907	The TURBO DNA-*free* Kit is ideal for removing contaminating DNA from RNA preparations. The kit employs Ambion® TURBO DNase (patent pending), a specifically engineered hyperactive DNase that exhibits up to 350% greater catalytic efficiency than wild type DNase I. It also includes a novel reagent for removing the DNase without the hassles or hazards of phenol extraction or alcohol precipitation—and without heat inactivation, which can cause RNA degradation.
TaqMan® DNA Template Reagents<br>P/N 401970	TaqMan® DNA Template Reagents provide the necessary components to perform a standard-dilution series of human DNA for quantitative real-time PCR. Use with the TaqMan® RNase P Detection Reagents (FAM™) Kit to generate a standard curve for quantifying the amount of DNA in your FFPE human DNA sample that is functional as a PCR template. For more information, go to www.invitrogen.com/site/us/en/home/support/technical-support.html.
TaqMan® RNase P Detection Reagents (FAM™) Kit<br>P/N 4316831	The TaqMan® RNase P Detection Reagents Kit provides the components needed to detect and quantitate genomic copies of the single-copy human RNase P gene using the 5' nuclease assay. Use with TaqMan® DNA Template Reagents to calculate the percentage of PCR-functional template in your FFPE human DNA sample.

II. RecoverAll Total Nucleic Acid Isolation Protocol

A. Before You Begin

RNase precautions

Lab bench, pipettors, microtome blade, and cutting surface

Before working with RNA, it is always a good idea to clean the lab bench, pipettors, and sectioning equipment with an RNase decontamination solution (e.g., Ambion® RNase*Zap*® Solution, P/N AM9780).

Gloves and RNase-free technique

Wear laboratory gloves for this procedure; they protect you from the reagents, and they protect the RNA from nucleases that are present on skin.

Use RNase-free pipette tips to handle the kit reagents, and avoid putting used tips into the reagent containers.

Prepare Wash Solutions

a. Add 42 mL of ACS grade 100% ethanol to the bottle labeled Wash 1 Concentrate. Mix well.

b. Add 48 mL of ACS grade 100% ethanol to the bottle labeled Wash 2/3 Concentrate. Mix well.

c. Cap the wash solution bottles tightly to prevent evaporation.

d. Mark the labels to indicate that the ethanol has been added.

The final solutions will be referred to as ***Wash 1*** and ***Wash 2/3*** in the procedure.

Protocol notes

If recovery of miRNA is an experimental goal, we recommend making slices 10 µm or thicker from the tissue block. Slices that are less than 10 µm thick typically yield a monolayer of cells, which have a greater risk of miRNA loss because cells are more likely to be split open. If the cytoplasm is exposed, loss of miRNA can occur during the deparaffinization washing steps. We have found that sections of at least 10 µm provide more than one cell thickness of the tissue and maximize recovery of miRNA.

B. Deparaffinization

1. Assemble FFPE sections equivalent to ≤ 80 µm or ≤ 35 mg unsectioned core

- Use samples that were cut or cored from the interior of the paraffin block, to minimize nucleic acid damaged by exposure to the atmosphere during storage.
- If you are preparing tissue sections, follow step 1a, then continue the protocol at step 2.
- If you are preparing unsectioned core samples, follow step 1b, then continue the protocol at step 2.

1a. Cut 5–20 µm FFPE sections to obtain the equivalent of ≤ 80 µm

a. Cut 5–20 µm sections from FFPE tissue blocks using a microtome.

NOTE
For recovery of miRNA, we recommend using ≥10 µm slices.

b. Place the equivalent of ≤ 80 µm of tissue slices (i.e., a maximum of four 20 µm, eight 10 µm, or sixteen 5 µm slices) in a 1.5 mL microcentrifuge tube.

1b. Grind ≤ 35 mg unsectioned FFPE samples in liquid nitrogen

a. Cut an unsectioned core sample, up to 35 mg, from a paraffin block.

b. Crush or grind the sample in liquid nitrogen or on dry ice, and place in a 1.5 mL microcentrifuge tube.

2. Add 1 mL 100% xylene, mix, and incubate for 3 min at 50°C

Xylene treatment completely removes paraffin from the sections.

a. Add 1 mL 100% xylene to the sample.

WARNING
Xylene is a toxic substance. Read the manufacturer's MSDS. Handle it only in a well ventilated area using personal protection equipment. Dispose of xylene waste according to applicable regulations.

b. Vortex briefly to mix.

c. Centrifuge briefly to bring any tissue that is stuck to the sides of the tube down into the xylene.

d. Heat the sample for 3 min at 50°C to melt the paraffin.

3. Centrifuge for 2 min at maximum speed, and discard the xylene

a. Centrifuge the sample for 2 min at room temperature and maximum speed to pellet the tissue.

b. (Optional) If the sample does not form a tight pellet, recentrifuge for an additional 2 min. If a tight pellet still does not form, then proceed with caution in step c.

c. Remove the xylene without disturbing the pellet. Discard the xylene.

NOTE
At this step, the tissue is usually clear and can be difficult to see. If the pellet is loose, you may need to leave some xylene in the tube to avoid removing any tissue pieces.

4. Wash the pellet twice with 1 mL 100% ethanol

The ethanol washes remove xylene from the sample and accelerate drying of the tissue.

a. Add 1 mL of 100% ethanol (room temperature) to the sample and vortex to mix.
The tissue should turn opaque.

b. Centrifuge the sample for 2 min at room temperature and maximum speed to pellet tissue.

c. Remove and discard the ethanol without disturbing the pellet.
The ethanol will contain trace amounts of xylene, and must be discarded accordingly.

d. Repeat steps a–c above to wash a second time with 1 mL of 100% ethanol.

e. Briefly centrifuge again to collect any remaining drops of ethanol in the bottom of the tube. Remove as much residual ethanol as possible without disturbing the pellet.

5. Vacuum or air dry the pellet to remove residual ethanol

Follow step 5a, to vacuum dry the pellet, or step 5b, to air dry the pellet, then continue the protocol at section *C. Protease Digestion*.

5a. Dry the pellet via vacuum centrifugation

Dry in a centrifugal vacuum concentrator at medium or low heat:

- Use medium heat (40–45°C) for ≤ 20 min.
- Use low heat (37–40°C) for 20–40 min.

5b. Air dry the pellet

Air dry for 15–45 min at room temperature.

NOTE

Larger tissue sections may not dry completely in 45 min. We recommend using a centrifugal vacuum concentrator for larger tissue sections.

C. Protease Digestion

1. Add Digestion Buffer and Protease

a. Use the following table to determine the appropriate amount of Digestion Buffer needed for your tissue sample.

Sample size	Digestion Buffer per sample
≤ 40 µm	100 µL
40–80 µm	200 µL

b. Add Digestion Buffer to each sample.

c. Add 4 µL Protease to each sample.

d. Swirl the tube gently to mix and to immerse the tissue. If tissue sticks to the sides of the tube, use a pipet tip to push it into the solution, or briefly centrifuge to bring the tissue down into the solution.

2. Incubate at 50°C/80°C (RNA) or at 50°C (DNA)

Follow step 2a, for RNA isolation, or step 2b, for DNA isolation, then continue the protocol at *D. Nucleic Acid Isolation*.

2a. RNA isolation: incubate for 15 min at 50°C, then 15 min at 80°C

For RNA isolation, incubate the sample in heat blocks for 15 min at 50°C, then 15 min at 80°C.

Extending the incubation at 80°C substantially (more than 2 min) may result in RNA degradation.

The sample mixture may not clarify after 30 min. Avoid removing undigested tissue pieces when applying to the Filter Cartridge in step D.3 on page 11.

2b. DNA isolation: incubate for 16 hr at 50°C

For DNA isolation, incubate the sample for 16 hr at 50°C.

Most sample mixtures will clarify after 16 hr. If the sample does not clarify, it may be heavily oxidized and therefore somewhat resistant to protease digestion. Samples that do not clarify may have slightly lower yields and smaller DNA fragments.

Increasing the incubation time at 50°C to up to 48 hr usually results in recovery of DNA with slightly increased functionality. This may be beneficial in downstream applications requiring larger PCR amplicons, or in sequencing or methylation analysis.

STOPPING POINT

Samples can be stored at –20°C, then thawed on ice before proceeding to D. Nucleic Acid Isolation.

D. Nucleic Acid Isolation

Protocol note

The RNA and DNA isolation procedures are identical with the exception of the nuclease treatment (step E.1 on page 11) and elution temperature (step E.4 on page 12).

1. Prepare Isolation Additive/ethanol mixture

Combine the indicated amounts of Isolation Additive and ethanol, according to the volume of Digestion Buffer used in your sample.

For multiple samples, prepare a master mix for all samples plus ~5% overage.

	Volume of Digestion Buffer	
	100 µL	200 µL
Isolation Additive	120 µL	240 µL
100% ethanol	275 µL	550 µL
Total	395 µL	790 µL

2. Add Isolation Additive/ethanol and mix

a. Add the appropriate volume of Isolation Additive/ethanol mixture to each sample.

b. Mix by pipetting up and down.
Some samples may appear white and cloudy after mixing.

3. Pass the mixture through a Filter Cartridge

a. For each sample, place a Filter Cartridge in one of the Collection Tubes supplied.

b. Pipet up to 700 μL of the sample/ethanol mixture (from step 2) onto the Filter Cartridge and close the lid.
To prevent clogging of the filter, avoid pipetting large pieces of undigested tissue onto the Filter Cartridge.

NOTE

Do not centrifuge Filter Cartridges at relative centrifugal forces greater than 10,000 x g; higher forces may damage the filters.

c. Centrifuge at 10,000 x g (typically 10,000 rpm) for 30 sec to pass the mixture through the filter.

d. Discard the flow-through, and re-insert the Filter Cartridge in the same Collection Tube.

e. If necessary, repeat steps a–d until all the sample mixture has passed through the filter.

4. Wash with 700 μL of Wash 1

a. Add 700 μL of Wash 1 to the Filter Cartridge.

b. Centrifuge for 30 sec at 10,000 x g to pass the mixture through the filter.

c. Discard the flow-through, and re-insert the Filter Cartridge in the same Collection Tube.

5. Wash with 500 μL of Wash 2/3, and then centrifuge to remove residual fluid

a. Add 500 μL of Wash 2/3 to the Filter Cartridge.

b. Centrifuge for 30 sec at 10,000 x g to pass the mixture through the filter.

c. Discard the flow-through, and re-insert the Filter Cartridge in the same Collection Tube.

d. Spin the assembly for an additional 30 sec to remove residual fluid from the filter.

E. Nuclease Digestion and Final Nucleic Acid Purification

Before you begin (DNA isolation)

For DNA isolation only, preheat Elution Solution or nuclease-free water to 95°C.
If you intend to vacuum dry your recovered nucleic acid sample, use nuclease-free water for elution in step 4 on page 12, because Elution Solution contains salts that, if concentrated, may affect downstream applications.

1. Add DNase or RNase mix to each Filter Cartridge and incubate for 30 min

Follow step 1a, for RNA isolation, or step 1b, for DNA isolation, then continue the protocol at step 2.

1a. RNA isolation: add 60 µL DNase mix and incubate for 30 min at room temp

a. Combine the following solutions to make the DNase mix (a master mix can be used if there is more than one sample).

Amount (per reaction)	Component
6 µL	10X DNase Buffer
4 µL	DNase
50 µL	Nuclease-free Water

b. Add 60 µL of the DNase mix to the ***center*** of each Filter Cartridge.

c. Cap the tube and incubate for 30 min at room temp (22–25°C).

1b. DNA isolation: add 60 µL RNase mix and incubate for 30 min at room temp

a. Combine the following solutions to make the RNase mix (a master mix can be used if there is more than one sample).

Amount (per reaction)	Component
10 µL	RNase A
50 µL	Nuclease-free Water

b. Add 60 µL of the RNase mix to the ***center*** of each Filter Cartridge.

c. Cap the tube and incubate for 30 min at room temperature (22–25°C).

2. Wash with 700 µL of Wash 1

a. Add 700 µL of Wash 1 to the Filter Cartridge.

b. Incubate for 30–60 sec at room temperature.

c. Centrifuge for 30 sec at 10,000 x g.

d. Discard the flow-through, and re-insert the Filter Cartridge in the same Collection Tube.

3. Wash twice with 500 µL of Wash 2/3, then centrifuge to remove residual fluid

a. Add 500 µL of Wash 2/3 to the Filter Cartridge.

b. Centrifuge for 30 sec at 10,000 x g.

c. Discard the flow-through, and re-insert the Filter Cartridge in the same Collection Tube.

d. Repeat steps a–c to wash a second time with 500 µL of Wash 2/3.

e. Centrifuge the assembly for 1 min at 10,000 x g to remove residual fluid from the filter.

4. Elute with 60 µL Elution Solution or nuclease-free water at room temp (RNA) or 95°C (DNA)

a. Transfer the Filter Cartridge to a fresh Collection Tube.

b. Apply 60 µL of Elution Solution or nuclease-free water to the ***center of the filter***, and close the cap.
- ***For RNA isolation,*** use room temperature eluant (22–25°C).
- ***For DNA isolation,*** use eluant preheated to 95°C.

IMPORTANT

The Elution Solution contains salts that, if concentrated, may affect downstream applications. If you intend to vacuum dry the sample, elute in nuclease-free water.

c. Allow the sample to sit at room temperature for 1 min.

d. Centrifuge for 1 min at maximum speed to pass the mixture through the filter.
   The eluate contains the RNA or DNA.

e. Store the nucleic acid at –20°C or colder.

f. (Optional) To store your sample for an extended period of time, or if a very small amount of nucleic acid was recovered, transfer the eluate to a non-stick tube (e.g., P/N 12450) to prevent loss of nucleic acid.

III. Assessing Nucleic Acid Yield and Integrity

A. Factors Affecting Nucleic Acid Recovery and Integrity

Tissue fixation and embedding causes nucleic acid modifications

During the tissue fixation process, formaldehyde reacts primarily with the nitrogen atoms of lysine, arginine, and histidine, resulting in extensive cross-linking of the protein matrix within each cell. Some of these crosslinks extend to the nucleic acid species present, primarily through the exocyclic nitrogens of adenine and cytidine. The subsequent embedding process further accelerates this chemistry by heating the sample, often in the formalin solution prior to the soak in hot paraffin.

The resulting extensive web of protein-protein and protein-nucleic acid crosslinks tends to physically entrap nucleic acid species, even those that are not chemically crosslinked. Protease treatment breaks down this web and frees the nucleic acid. Even after protease treatment, some of the nucleic acid bases will still be crosslinked to amino acids, peptides, or other biomolecules. There may also be other, unidentified, formaldehyde-induced modifications. Any sites of modification on the nucleic acid chain provide a block to polymerases using it as a template.

The degree of nucleic acid fragmentation varies

Nucleic acid modifications also contribute to the fragmentation of RNA (DNA to a lesser extent) over time, even while stored in the block of paraffin. For this reason, the average size of RNA obtained from FFPE samples can vary widely. One key factor that contributes to RNA quality appears to be removal of all excess formaldehyde during the original embedding procedure. Another is storage of blocks without cut faces, which prevents damage from atmospheric oxygen, water and other environmental factors such as light and infestation (fungus, insects, etc.).

With FFPE samples stored a relatively short time, broad peaks reminiscent of 18S and 28S rRNAs will be seen on electrophoretic analysis (see Figure 2 on page 17). Even with these samples, the extent of modification is apparent from the broadness of the peaks and a diminished function as a template for polymerases; quantitative PCR (RT or standard) will often show an increase in the cycle threshold of 5–6 cycles (with equivalent mass inputs) compared to RNA or DNA from unfixed tissue.

In older FFPE samples, the RNA is typically already highly fragmented, often with peak sizes of only 100 nt or so. In these cases, a “good” prep will merely obtain these fragments.

DNA recovered from FFPE samples tends to be much less fragmented, but still exhibits a broad size distribution, and again has diminished capacity to serve as a template for polymerases.

B. Assessing RNA Yield and Integrity

RNA yield

The expected RNA yield varies greatly depending on the tissue type and the method used for fixing and storing the sample. In general, RNA extraction from fixed, embedded tissues will yield about 50% of that obtained from unfixed tissue from the same source.

Spectrophotometry

The concentration of an RNA solution can be determined by measuring its absorbance at 260 nm. In-house scientists recommend using the NanoDrop 1000 Spectrophotometer because it is extremely quick and easy to use; measure 1.5 µL of the RNA sample directly.

Alternatively, the RNA concentration can be determined by diluting an aliquot of the preparation in TE (10 mM Tris-HCl, pH 8, 1 mM EDTA) and reading the absorbance in a traditional spectrophotometer at 260 nm. An A_{260} of 1 is equivalent to 40 µg RNA/mL, in a spectrophotometer with a 1 cm path length. Calculate the RNA concentration (µg/mL) as follows:

$$A_{260} \times \text{dilution factor} \times 40\ \mu g/mL = \mu g\ RNA/mL$$

Note that any contaminating DNA in the RNA prep will lead to an overestimation of yield, since all nucleic acids absorb at 260 nm.

Fluorometry

If a fluorometer or a fluorescence microplate reader is available, Molecular Probes' RiboGreen® fluorescence-based assay for RNA quantitation is a convenient and sensitive way to measure RNA concentration. Follow the manufacturer's instructions for using RiboGreen.

RNA quality

Spectrophotometry

The A_{260}/A_{280} ratio of the RNA is an indication of its purity. The RNA isolated with this protocol should have an A_{260}/A_{280} ratio of 1.8–2.1.

Agarose gel electrophoresis

You can also assess the quality of your RNA sample by agarose gel electrophoresis. For more information, go to www.invitrogen.com/site/us/en/home/support/technical-support.html.

RNA recovered from FFPE samples will typically appear smeared. In higher quality preparations, two broad bands representing 18S and 28S rRNA will be seen; only a smear will be visible in lower quality preparations.

Microfluidics analysis

Agilent's 2100 bioanalyzer used in conjunction with an RNA LabChip® Kit provides a powerful and sensitive method to assess RNA integrity. To use this system, follow the instructions for RNA analysis provided with the RNA LabChip Kit.

The data will mimic that seen on agarose gels. Higher quality preparations will show a mass range "mound" with peaks at the approximate size of the 18S (and sometimes 28S) rRNA, as in Figure 2. Poorer quality preparations will range in size from 80–350 nt, with a peak size of 80–100 nt.

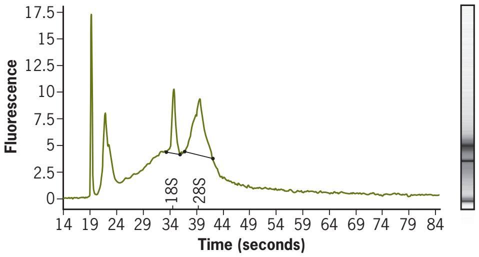

Figure 2. RNA prepared from FFPE mouse liver

RNA was isolated from 20 µm sections of FFPE mouse liver, after 2 months in storage at room temperature, using the RecoverAll Total Nucleic Acid Isolation Kit. The data were generated by running a sample of the preparation on an Agilent 2100 bioanalyzer using an RNA LabChip Kit.

Recovery of miRNA

Fragmentation of FFPE RNA seems to have a size endpoint of ~80 nt, and modifications affect only about 1% of the nucleotides. Thus, miRNA molecules, which are 21–23 nt, are basically untouched by the fixation/embedding process. Using slices that are ≥10 µm thickness and minimizing time in the xylene and ethanol washes will minimize loss of miRNA.

Downstream applications

The purity of recovered nucleic acid is sufficient for most applications. Nucleic acid fragmentation and some chemical modifications, caused by sample fixation and storage, will remain after the RecoverAll Total Nucleic Acid Isolation procedure. Therefore, some downstream procedures may need to be modified for best results. See section *A. Factors Affecting Nucleic Acid Recovery and Integrity* on page 15 for more detailed discussion.

Real-time RT-PCR

The RNA recovered with the RecoverAll Total Nucleic Acid Isolation Kit can be used in real-time RT-PCR analysis. Because the RNA extracted from fixed tissues is likely to be degraded, plan to analyze small amplicons.

RNA from FFPE samples always require more rounds of PCR to detect signal than that from frozen tissues from the same source. This is probably due to nucleic acid modifications incurred by the fixation process.

Array analysis

While RNA recovered with the RecoverAll Total Nucleic Acid Isolation Kit can be amplified for use in array analysis using, for example, the MessageAmp™ III or MessageAmp™ Premier Kits (P/N AM1793, AM1792, respectively), there are several caveats. Since amplification procedures prime from the 3' end of mRNAs, the fragmentation and presence of formaldehyde-induced lesions on the RNA recovered with this kit will create a population of targets that are weighted to the extreme 3' ends of the messages. By using appropriate probes on the arrays, or by applying a filter to the data so that only 3' end-most probes are analyzed, biologically relevant information can be obtained. Even with these compensatory steps, older samples (archived for ≥10 yr) will often be so highly fragmented that the number of expressed genes called present will be drastically reduced.

C. DNA Yield and Quality

DNA yield

DNA yield and quality depends on the tissue and fixing procedure. See section *A. Factors Affecting Nucleic Acid Recovery and Integrity* on page 15 for a discussion. Using the RecoverAll Total Nucleic Acid Isolation Kit, we have recovered 0.1–3.5 µg DNA per mg FFPE tissue sample.

The concentration of a DNA solution can be determined by measuring its absorbance at 260 nm. In-house scientists recommend using the NanoDrop 1000 Spectrophotometer because it is extremely quick and easy to use; measure 1.5 µL of the DNA sample directly.

Alternatively, determine the DNA concentration by diluting an aliquot of the preparation in TE and reading the absorbance at 260 nm in a traditional spectrophotometer. An A_{260} of 1 is equivalent to 50 µg double-stranded DNA/mL, in a spectrophotometer with a 1 cm path length. Calculate the DNA concentration (µg/mL) as follows:

$$A_{260} \times \text{dilution factor} \times 50\ \mu g/mL = \mu g\ DNA/mL$$

DNA quality

Spectrophotometry

The A_{260}/A_{280} ratio of the DNA is an indication of its purity. The DNA isolated with this protocol should have an A_{260}/A_{280} ratio of 1.7–1.9.

Quantitative real-time PCR vs a DNA standard

The ability of recovered DNA to function as a template for PCR can be measured using the TaqMan® DNA Template Reagents Kit (P/N 401970) and the TaqMan® RNase P Detection Reagents Kit (P/N 4316831). For more information, go to www.invitrogen.com/site/us/en/home/support/technical-support.html.

Agarose gel electrophoresis

The DNA obtained from this procedure will always be a population of sheared fragments. You can assess the average size of this population using standard electrophoretic methods. This will require the use of ~1 µg of DNA, which may be a sizeable fraction of the yield for some samples.

Microfluidics analysis

A bioanalyzer, such as the Agilent 2100, requires a much smaller amount of the recovered DNA than does agarose gel electrophoresis. The recovered DNA typically appears as a broad "smear," covering a size range up to thousands of base pairs, with the modal size varying from less than 100 bp to ~3000 bp, depending on the age and condition of the original sample.

Downstream applications

DNA recovered with the RecoverAll Total Nucleic Acid Isolation Kit can be used in PCR analysis and other downstream applications; however, the same caveats apply as for analysis of RNA. Modifications from the fixation and embedding process occur on average every 100–200 bp; therefore, we recommend analysis of amplicons around 100 bp or smaller.

IV. Troubleshooting

A. Nucleic Acid Appears Fragmented

Nucleic acid was degraded before extraction

Sample fixation, embedding, and storage have a strong impact on the integrity of nucleic acid, especially of RNA, in FFPE tissue samples. Some samples simply do not contain large RNA molecules. Please see section *III.A. Factors Affecting Nucleic Acid Recovery and Integrity* on page 15 for further discussion.

Overheating during protease digestion

Overheating during the protease digestion step (II.C.2 on page 9) can result in degradation of the RNA. Do not extend the 15 min incubation at 80°C more than 2 min. Make sure the temperature of the heating device used does not fluctuate significantly.

RNA is degraded after purification

Once the RNA is isolated, it is sensitive to nucleases, so normal handling precautions for RNA are required.

B. Yield of Nucleic Acid Is Low

Low RNA yield can be due to tissue type

Tissues can vary enormously in their RNA content and in extraction efficiency. In addition, very fibrous tissues, such as muscle, will tend to form a more tightly-crosslinked web upon fixation, so relative recovery of RNA will be lower.

Sample is oxidized

If the suspension does not clarify during protease digestion (step II.C.2 on page 9), this could indicate that it is oxidized. Samples that do not clarify may have slightly lower yields and smaller RNA fragments. In most cases, usable nucleic acid can still be extracted from these samples.

Yields of RNA may be improved slightly by extending the protease digestion time at 50°C by 30 min to 3 hr. The incubation time at 80°C should not be extended by more than 2 min.

Downstream functionality of recovered DNA is low

The ability of recovered DNA to function as a template for PCR can be measured using the TaqMan® DNA Template Reagents Kit (P/N 401970) and the TaqMan® RNase P Detection Reagents Kit (P/N 4316831). For more information, go to www.invitrogen.com/site/us/en/home/support/technical-support.html.

Increasing the incubation time at 50°C (step II.C.2b on page 10) to up to 48 hr usually results in recovery of DNA with slightly increased functionality. This may be beneficial in downstream applications requiring larger PCR amplicons, or in sequencing or methylation analysis.

Poor elution from the Filter Cartridge

It is important to allow the sample to sit for one full minute in step II.E.4 on page 12 of the protocol to allow the Elution Solution or other eluant to permeate the entire filter before centrifugation.

The room temperature elutant should be at 22–25°C.

C. Nucleic Acid Inhibits Enzymatic Reactions; Contaminants in Nucleic Acid

DNA contamination of RNA

Poor DNase digestion in step II.E.1a on page 12 can be due to too low a digestion temperature. If the room temperature of your laboratory is not 22–25°C, use a heat block or incubator to maintain this temperature.

Further digest the prep with TURBO™ DNase (RNase-free)
Contaminating DNA in the recovered RNA can be removed by DNase digestion, with subsequent removal of DNase and buffer from the sample. We recommend the TURBO DNA-*free*™ Kit (P/N 1907), which is formulated specifically for this type of application.

Other contaminants

Ethanol precipitation can further purify the recovered nucleic acid.

a. Add ammonium acetate to a final concentration of 2–2.5 M and mix well.

b. Add 4 volumes of ethanol, and mix again.

c. Chill at –20°C or lower for at least 30 min.

NOTE
To maximize miRNA recovery, chill at –20°C for ≥12 hr.

d. Centrifuge at 16,000 x g for 20–30 min to pellet the RNA.

e. Wash the pellet twice with 80% ethanol.

f. Resuspend the RNA in nuclease-free water or TE (10 mM Tris-HCl, pH 8, 1 mM EDTA).

V. Appendix

A. References

Cronin M, Pho M, Dutta D, Stephans JC, Shak S, Kiefer MC, Esteban JM, Baker JB (2004) Measurement of gene expression in archival paraffin-embedded tissues: development and performance of a 92-gene reverse transcriptase-polymerase chain reaction assay. *Am J Pathol.* **164**(**1**):35–42.

Masuda N, Ohnishi T, Kawamoto S, Monden M, Okubo K (1999) Analysis of chemical modification of RNA from formalin-fixed samples and optimization of molecular biology applications for such samples. *Nucleic Acids Res*. **27**(**22**):4436–4443.

Specht K, Richter T, Muller U, Walch A, Werner M, Hofler H (2001) Quantitative gene expression analysis in microdissected archival formalin-fixed and paraffin-embedded tumor tissue. *Am J Pathol.* **158**(**2**):419–429.

B. Quality Control

Functional testing

RNA and DNA are isolated from embedded tissue following the protocol described in section *II. RecoverAll Total Nucleic Acid Isolation Protocol* starting on page 7. RNA and DNA are checked for minimal yield requirements and suitability for real-time RT-PCR.

Nuclease testing

Relevant kit components are tested in the following nuclease assays:

RNase activity
Meets or exceeds specification when a sample is incubated with labeled RNA and analyzed by PAGE.

Nonspecific endonuclease activity
Meets or exceeds specification when a sample is incubated with supercoiled plasmid DNA and analyzed by agarose gel electrophoresis.

Exonuclease activity
Meets or exceeds specification when a sample is incubated with labeled double-stranded DNA, followed by PAGE analysis.

C. Safety Information

WARNING

GENERAL SAFETY. *Using this product in a manner not specified in the user documentation may result in personal injury or damage to the instrument or device. Ensure that anyone using this product has received instructions in general safety practices for laboratories and the safety information provided in this document.*

- *Before using an instrument or device, read and understand the safety information provided in the user documentation provided by the manufacturer of the instrument or device.*

- *Before handling chemicals, read and understand all applicable Safety Data Sheets (SDSs) and use appropriate personal protective equipment (gloves, gowns, eye protection, etc). To obtain SDSs, see the "Documentation and Support" section in this document.*

1. Chemical safety

WARNING

GENERAL CHEMICAL HANDLING. *To minimize hazards, ensure laboratory personnel read and practice the general safety guidelines for chemical usage, storage, and waste provided below, and consult the relevant SDS for specific precautions and instructions:*

- *Read and understand the Safety Data Sheets (SDSs) provided by the chemical manufacturer before you store, handle, or work with any chemicals or hazardous materials. To obtain SDSs, see the "Documentation and Support" section in this document.*
- *Minimize contact with chemicals. Wear appropriate personal protective equipment when handling chemicals (for example, safety glasses, gloves, or protective clothing).*
- *Minimize the inhalation of chemicals. Do not leave chemical containers open. Use only with adequate ventilation (for example, fume hood).*
- *Check regularly for chemical leaks or spills. If a leak or spill occurs, follow the manufacturer's cleanup procedures as recommended in the SDS.*
- *Handle chemical wastes in a fume hood.*
- *Ensure use of primary and secondary waste containers. (A primary waste container holds the immediate waste. A secondary container contains spills or leaks from the primary container. Both containers must be compatible with the waste material and meet federal, state, and local requirements for container storage.)*
- *After emptying a waste container, seal it with the cap provided.*
- *Characterize (by analysis if necessary) the waste generated by the particular applications, reagents, and substrates used in your laboratory.*
- *Ensure that the waste is stored, transferred, transported, and disposed of according to all local, state/provincial, and/or national regulations.*
- ***IMPORTANT!*** *Radioactive or biohazardous materials may require special handling, and disposal limitations may apply.*

2. Biological hazard safety

WARNING

Potential Biohazard. *Depending on the samples used on the instrument, the surface may be considered a biohazard. Use appropriate decontamination methods when working with biohazards.*

WARNING

BIOHAZARD. *Biological samples such as tissues, body fluids, infectious agents, and blood of humans and other animals have the potential to transmit infectious diseases. Follow all applicable local, state/provincial, and/or national regulations. Wear appropriate protective equipment, which*

includes but is not limited to: protective eyewear, face shield, clothing/lab coat, and gloves. All work should be conducted in properly equipped facilities using the appropriate safety equipment (for example, physical containment devices). Individuals should be trained according to applicable regulatory and company/institution requirements before working with potentially infectious materials. Read and follow the applicable guidelines and/or regulatory requirements in the following:

In the U.S.:

- *U.S. Department of Health and Human Services guidelines published in Biosafety in Microbiological and Biomedical Laboratories found at: www.cdc.gov/biosafety*
- *Occupational Safety and Health Standards, Bloodborne Pathogens (29 CFR§1910.1030), found at: www.access.gpo.gov/nara/cfr/waisidx_01/29cfr1910a_01.html*
- *Your company's/institution's Biosafety Program protocols for working with/handling potentially infectious materials.*
- *Additional information about biohazard guidelines is available at: www.cdc.gov*

In the EU:

Check local guidelines and legislation on biohazard and biosafety precaution and refer to the best practices published in the World Health Organization (WHO) Laboratory Biosafety Manual, third edition, found at: www.who.int/csr/resources/publications/biosafety/WHO_CDS_CSR_LYO_2004_11/en/

VI. Documentation and Support

A. Obtaining SDSs

Safety Data Sheets (SDSs) are available from:

www.invitrogen.com/sds

or

www.appliedbiosystems.com/sds

Note: For the SDSs of chemicals not distributed by Life Technologies, **contact the chemical manufacturer.**

B. Obtaining support

For the latest services and support information for all locations, go to:

www.invitrogen.com

or

www.appliedbiosystems.com

At the website, you can:

- Access worldwide telephone and fax numbers to contact Technical Support and Sales facilities
- Search through frequently asked questions (FAQs)
- Submit a question directly to Technical Support
- Search for user documents, SDSs, vector maps and sequences, application notes, formulations, handbooks, certificates of analysis, citations, and other product support documents
- Obtain information about customer training
- Download software updates and patches

1975MC

Headquarters
5791 Van Allen Way | Carlsbad, CA 92008 USA
Phone +1 760 603 7200 | Toll Free in USA 800 955 6288
For support visit www.appliedbiosystems.com/support

www.lifetechnologies.com

life technologies™
